# EBV Viral Loads in Diagnosis, Monitoring, and Response Assessment

**DOI:** 10.3389/fonc.2019.00062

**Published:** 2019-02-12

**Authors:** Hiroshi Kimura, Yok-Lam Kwong

**Affiliations:** ^1^Department of Virology, Nagoya University Graduate School of Medicine, Nagoya, Japan; ^2^Department of Medicine, Queen Mary Hospital, Hong Kong, China

**Keywords:** EBV-DNA, chronic active EBV infection, extranodal NK/T-cell lymphoma, nasal type, post-transplant lymphoproliferative disorders, Hodgkin lymphoma

## Abstract

The quantification of circulating Epstein Barr virus (EBV) DNA loads has played an important role in the diagnosis and management of EBV-associated lymphoid malignancies. Viral load measurement is particularly useful for monitoring EBV-DNA in hematopoietic stem cell transplant patients, and for assessing the prognosis or response to therapy of EBV-associated intractable lymphomas like extranodal NK/T-cell lymphoma, nasal type. Cell-free EBV-DNA in plasma can be used as a biomarker for estimating the severity or prognosis of these lymphomas. In addition to plasma, whole blood has been used for the management of transplant patients. Although measuring EBV-DNA has been useful, there is a lack of standardization and the optimal specimens for measuring viral loads are unknown. This can be attributed to the different forms of EBV-DNA that exist in peripheral blood and the different pathologies that result from diverse EBV disease states. As a result, guidelines for EBV diagnosis or the initiation of treatment are unclear. However, the newly established World Health Organization standard for EBV quantification will encourage collaborative studies across institutions and countries to establish proper guidelines for EBV diagnosis and the initiation of treatment.

## Introduction

Epstein Barr virus (EBV) is a ubiquitous tumor virus that belongs to the gammaherpesvirus subfamily. EBV is associated with a variety of lymphomas/leukemias, and epithelial malignancies, including nasopharyngeal carcinoma (NPC), lymphoepithelioma-like carcinoma, and gastric cancer (1). The diagnosis of EBV-associated malignancies is principally based on biopsy of the primary tumor. However, it can be challenging to perform a biopsy because of poor patient status or difficulties accessing the tumor.

A non-invasive and more convenient method of EBV diagnosis would be the quantification of EBV viral loads in peripheral blood. In fact, EBV viral load quantification has recently played a more important role in the diagnosis and management of EBV-associated diseases (2, 3). Additionally, measuring viral loads is particularly useful for monitoring EBV-DNA in transplant patients with risks of EBV-associated post-transplant lymphoproliferative disease (PTLD), and assessing the response to therapy of these malignancies.

In this review, we first summarize the principles behind the quantification of viral loads based on the pathophysiology of EBV infections. We then introduce applications of viral load measurements for EBV-associated diseases with a focus on lymphoid malignancies, including lymphoma, leukemia, and lymphoproliferative diseases (LPD) (4, 5).

## Pathophysiology of EBV Infection and Principles of EBV Load Measurement

**Figure 1:**
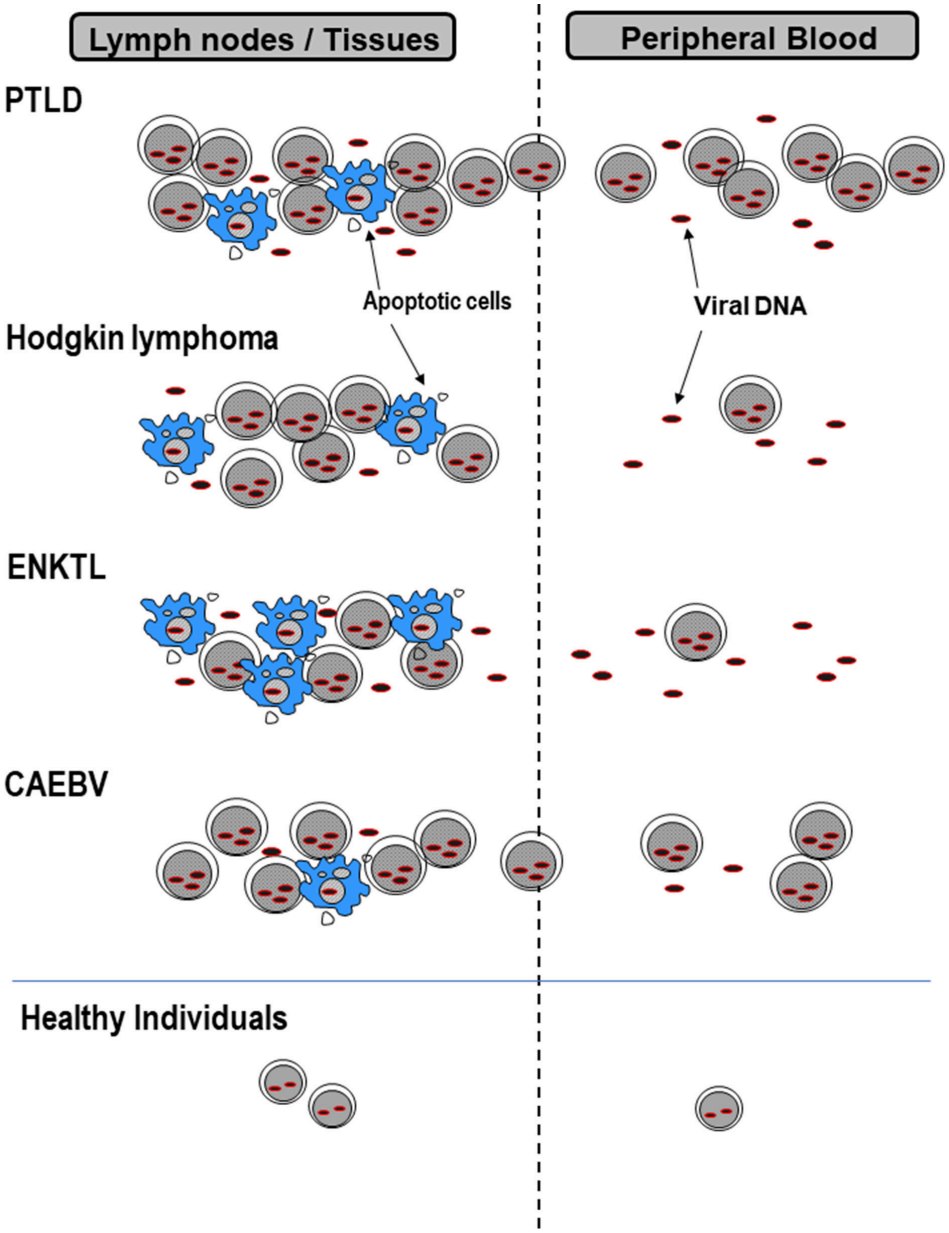
EBV forms that exist in lymph nodes or peripheral blood. EBV-infected cells or cell-free EBV-DNA deriving from apoptotic cells in lymph nodes or tissues, pass into peripheral blood. The amount of cell-associated or cell-free EBV-DNA differs among diseases, and therefore the best specimens for measuring viral loads are also different. PTLD, post-transplant lymphoproliferative disorders; ENKTL, extranodal NK/T-cell lymphoma, nasal type; CAEBV, chronic active EBV infection.

During primary infection, EBV attaches to B cells via the binding of EBV gp350/220 to CD21 and gH/gL/gp42 to HLA class II molecules on the cell surface. The binding of the two viral proteins to its receptors allow entry of EBV into B cells, establishing thereafter a life-long infection (1). Following primary infection, EBV persists latently in memory B cells at a low viral level (~1 in 10,000 to 100,000 B cells) (6). Therefore, even healthy individuals can carry measurable EBV loads in their peripheral blood. In addition to B cells, EBV can infect T or natural killer (NK) cells, although the exact mechanism whereby EBV infects T or NK cells is unknown (7). EBV exists in the nucleus of these lymphocytes in an episomal form and latently infects the cell without virus production. In EBV-associated lymphoid malignancies, EBV-infected lymphoma cells move into the circulation and can be detected in the peripheral blood (2). However, their inflow to peripheral blood depends on the expression patterns of extracellular adhesion molecules and differs among lymphoma types (Figure 1). For example, in post-transplant lymphoproliferative disorders (PTLD), EBV-infected cells proliferate in lymphoid tissues and transit into the peripheral blood. Therefore, most of the viral DNA in the peripheral blood is cell-associated. Yet, in Hodgkin lymphoma (HL) most EBV-infected lymphoma cells remain in tissues and the episomal EBV-DNA derived from apoptotic or necrotic cells passes into the peripheral blood. Consequently, the EBV-DNA found in the blood in HL is largely cell-free. Cell-free EBV-DNA can therefore indicate a patient's tumor burden and the cell damage caused by inflammation or immunity. Therefore, cell-free DNA can be used as a biomarker for assessing disease severity or the prognosis of patients (3). In NPC (not described in detail in this review), measurements of circulating EBV-DNA are used for staging, predicting outcomes, and even for screening early asymptomatic patients (8–10). However, optimal specimens for measuring viral loads are different among diseases. This can be attributed to the different forms of EBV-DNA that exist in peripheral blood and the different pathologies that result from diverse EBV disease states (Figure 1). The application of EBV load measurement to representative EBV-associated lymphoid malignancies is outlined in Table 1.

**Table 1:** EBV-associated lymphoid malignancies and applications of measuring EBV viral loads.

	**Association to EBV**	**Infected cells**	**Viral load measurement**	
			**Purpose**	**Specimens**
PTLD	>90%	B	Monitoring; treatment response	Plasma/whole blood
Hodgkin lymphoma	~75% (mixed cellularity and lymphocyte-depleted type)	Hodgkin/Reed-Sternberg cells	Prognostic evaluation; treatment response	Plasma
ENKTL	100%	NK, T	Prognostic evaluation; treatment response	Plasma/whole blood
CAEBV	100%	T, NK, (B)	1) Diagnosis 2) Prognostic evaluation	1) PBMCs/whole blood 2) Plasma

*PTLD, post-transplant lymphoproliferative disorders; ENKTL, extranodal NK/T-cell lymphoma, nasal type; CAEBV, chronic active EBV infection; PBMCs, peripheral blood mononuclear cells*.

To measure EBV loads, real-time polymerase chain reaction (PCR) is a standard and widely-used method (8, 11). The real-time PCR method measures the accumulation of amplified products with a laser scanning in a closed tube or 96-well plate format (12). Fluorogenic probes and SYBR green I dye are used as markers for accumulation of PCR products. This method is rapid, sensitive, reproducible, and is advantageous because the reaction is performed in closed tubes or wells, thereby reducing the risk of carry-over contamination (2). The main drawback of real-time PCR is that there are no standard protocols, kits, or machines. Facilities that quantify EBV-DNA use their own “homemade” system or commercially available kits that have been modified, employing different primer/probe designs, standards, and equipment. Therefore, the comparison of values across laboratories and countries has been difficult. Consequently, the cut-off values used for the diagnosis of EBV or the initiation of treatment vary among institutions. Standardization is necessary to establish guidelines.

Recently, the World Health Organization (WHO) International Standard for EBV was developed based on the results of a worldwide collaborative study group, and was released for the standardization of quantitative PCR (13). With this standard, comparisons across institutions will become easier and lead to the establishment of guidelines for the management of EBV-associated diseases.

A recently developed droplet digital PCR utilizes water-oil emulsion droplets that form the partitions separating template DNA (14, 15). The advantages of this method include extremely high sensitivity and absolute quantification without standard curves. This method is particularly useful for creating or evaluating accurate viral reference standards (16). Digital PCR is now increasingly applied for quantifying viral loads including EBV (17–20). With different types of fluorogenic probes, multiplex PCR to detect several viruses has been developed (21). This method is suitable in quantifying very low amount of virus or detecting mutated viruses because of the robustness to detect mismatches between mutated viruses and the primer-prove set (18). In fact, digital PCR was successfully used for the detection of low amount of virus in the aqueous humor (20). On the other hand, disadvantages of the method are high initial costs of equipment, relatively low throughput, and narrow dynamic ranges, compared with real time PCR (15, 22). It might be difficult to apply digital PCR for quantifying high viral load in peripheral blood such as EBV in patients with PTLD or hepatitis B virus in patients with chronic hepatitis. Future technological improvement will doubtlessly overcome these problems.

## Applications of Measuring EBV Loads for Different EBV-associated Lymphomas

### PTLD

PTLD, a subtype of immunodeficiency-associated LPD, is defined as lymphoid or plasmacytic proliferation that develops as a consequence of immunosuppression after solid organ allografting or hematopoietic stem cell transplantation (HSCT) (23, 24). Most PTLD are associated with EBV infection, but they constitute a spectrum ranging from EBV-driven polyclonal proliferation to EBV-positive or -negative proliferation that is indistinguishable from lymphomas occurring in immunocompetent individuals.

The diagnosis of PTLD is performed based on symptoms and/or signs consistent with PTLD, together with histological features. To associate EBV with a PTLD diagnosis, EBV-encoded small RNA (EBER) *in situ* hybridization or viral antigen detection is performed. EBV-DNA detection in peripheral blood is not enough to prove the diagnosis of EBV-PTLD. However, measuring EBV-DNA has been used to diagnose PTLD when biopsy samples cannot be obtained. EBV-DNA measurement has also been applied to monitoring viral loads in high-risk HSCT patients (25). European and American guidelines recommend prospective screening for EBV-DNA by quantitative PCR after allogeneic HSCT in cases at high-risk for EBV-PTLD (26, 27).

Measuring viral loads also allows a preemptive reduction in immunosuppression if possible, as the first part of patient management. These guidelines also moderately recommend that significant amounts of EBV-DNA without clinical symptoms of EBV disease are an indication for preemptive therapy. Because most PTLDs are of B-cell lineage and express CD20, preemptive treatment with rituximab in patients with rising EBV DNA is recommended. However, there are no consensus guidelines regarding the threshold of EBV DNA that warrants further work-up or preemptive therapy (28). There is also no consensus regarding a preference for specimens. According to the 2016 European guidelines, whole blood, plasma, and serum are appropriate specimens for monitoring EBV loads (27). Nevertheless, plasma EBV has been reported to be a better measure than cell-associated EBV from peripheral blood mononuclear cells (PBMCs) (29). There is still controversy between plasma and whole blood in terms of superiority for EBV-DNA monitoring (27, 30). In general, high sensitivity but low specificity is noted when whole blood is used for monitoring the EBV load in HSCT recipients. In patients with symptomatic PTLD, EBV-DNA was not detected in all plasma samples, whereas all whole blood specimens were positive for viral DNA (31). These results suggest that whole blood is a better source for the diagnosis of PTLD. On the contrary, plasma EBV-DNA declines or becomes undetectable in patients who respond to therapy, and therefore could be useful for response monitoring (29).

It should be emphasized that there is a difference between patients who have had solid organ allografting and HSCT patients (24). Immunosuppressive treatments in solid organ allograft recipients are modest compared to HSCT recipients who receive more severe immunosuppressive treatment. Correlations between higher EBV loads and the development of PTLD are seen in solid organ allograft recipients, but these correlations do not indicate high positive and negative predictive values (32). There is considerable overlap between the EBV loads in patients with PTLD and those in patients without PTLD. Furthermore, solid organ allograft recipients receive lifelong immunosuppression, so that there is a long-term risk of EBV-PTLD. Therefore, routine surveillance for EBV-DNA by quantitative PCR is not recommended in adult recipients (33). In children at high risks of primary EBV infection, routine surveillance is useful for the preemptive identification of patients at high risk of PTLD (33). Solid organ allograft recipients also sometimes carry chronic high EBV loads without symptoms consistent with PTLD (33, 34), but the significance of a high EBV load in terms of long-term health is unknown.

### HL

HL is a monoclonal lymphoid neoplasm composed of Hodgkin/Reed-Sternberg cells, which are derived from B cells in a background non-neoplastic reactive immune cells (35). Classic HL consists of four histological subtypes, and the association with EBV varies across subtypes. Among them, EBV is most commonly positive in mixed cellular HL and lymphocyte-depleted HL (Table 1). The diagnosis of HL is mainly based on histological features, and EBER *in situ* hybridization is used to determine if there is an association with EBV (36).

In patients with classic HL, very few of the EBV-DNA in plasma is encapsidated (37), suggesting that cell-free EBV-DNA is derived from apoptotic or necrotic EBV-infected cells in tumors (Figure 1). EBV-DNA detection in plasma is highly specific for EBV-positive HL and seems promising as a prognostic marker and an indicator of treatment responses (3). In fact, EBV-DNA in plasma is highly correlated with EBV tumor status in HL and is significant for determining the prognosis before therapy and at follow-up after 6 months (38).

### Extranodal NK/T-Cell Lymphoma, Nasal Type (ENKTL)

ENKTL is a predominantly extranodal lymphoma of T or NK cells, which is characterized by necrosis, a cytotoxic phenotype, and vascular damage or destruction (39). Most cases of ENKTL are genuine NK cell neoplasms, but some are of the T cell lineage. The diagnosis of ENKTL is made using histological features. Since ENKTL has an almost universal association with EBV infection in the lymphoma cells, the detection of EBER by *in situ* hybridization is important.

EBV-DNA levels in peripheral blood are a surrogate biomarker of tumor loads (40) and is used in making a diagnosis (41, 42). The quantification of EBV loads is also useful for prognostic assessment and the evaluation of treatment responses (43–46). A prognostic stratification model was proposed based on an international multicenter analysis (47). Both plasma and whole blood have been used for quantifying circulating EBV-DNA, and results from plasma and whole blood correlated with each other (44). Since there are few direct comparisons between plasma and whole blood, the better source is unclear. However, plasma appears to be a better biomarker for evaluating prognosis since plasma has several advantages over whole blood in terms of materials (3, 40). EBV-DNA in plasma is a better indicator of prognosis than EBV-DNA in PBMCs (43).

### Chronic Active EBV Infection (CAEBV)

CAEBV is a systemic EBV-positive polyclonal, oligoclonal, or (often) monoclonal lymphoproliferative disease characterized by fever, lymphadenopathy, hepatosplenomegaly, pancytopenia, interstitial pneumonitis, and skin involvement (hypersensitivity to mosquito bites or hydroa vacciniforme) (4, 48). In East Asians, EBV-infected cells are exclusively T or NK cells, while they are B or T cells in the Western hemisphere (49). CAEBV, T/NK cell type is an EBV-associated T/NK-cell LPD in childhood according to the 2017 WHO lymphoma classification (50). The diagnosis of CAEBV is based on: (1) infectious mononucleosis-like symptoms lasting >3 months, (2) increased EBV-DNA in peripheral blood or the demonstration of EBER in affected tissues, and (3) the exclusion of known immunodeficiencies, malignancies, or autoimmune disorders (48, 51). Since histological samples are not always obtained because of a lack of appropriate lesions for biopsies, the quantification of EBV-DNA in peripheral blood is necessary for its diagnosis. Monitoring for EBV-DNA is also useful for assessing the treatment response (34).

Although there is no current consensus regarding the optimal component to measure in peripheral blood, PBMCs have been used as a measure of diagnostic EBV load values for CAEBV (2, 51). EBV loads in PBMCs and those in plasma/serum correlate with each other to some extent, but inconsistent results (viral DNA is positive in PBMCs, but negative in plasma) have been seen in some patients, indicating that the EBV load in PBMCs is better than that in plasma for diagnostic purposes (52, 53). Whole blood, which contains both cell-associated and cell-free EBV-DNA, may be utilized for diagnosis as well. However, there are no comparison data from large populations. On the other hand, plasma EBV loads were higher at diagnosis in patients who were deceased compared to patients that survived, suggesting that cell-free EBV-DNA has prognostic value (34). The discrepancy between PBMCs and plasma is unclear, but EBV-DNA in plasma is derived from damaged virus-infected cells in organs and may reflect organ involvement.

## Future Directions

Real-time PCR was first applied to quantify EBV-DNA loads 20 years ago (8, 11). During the past two decades, evidence regarding the monitoring of viral DNA has accumulated in a variety of EBV-associated diseases. Determining a cut-off line to differentiate healthy individuals and those with malignancies is necessary. A lot of efforts have been devoted to establishing the cut-off value, but so far a consensus had not been reached (2, 26, 27, 54, 55). The lack of standardization has prevented institutions from collaborating with each other and has delayed consensus on standard guidelines. The release of the WHO standard for EBV quantification will boost collaborative studies across institutions and countries (13). Prospective studies using the WHO international standardized assay are necessary to establish a threshold at which preemptive therapy should be started in patients who have undergone HSCT. Chronic high EBV loads in solid organ allograft recipients should also be clarified to better manage these patients. Determining the preferred blood component for measurements is also an urgent task.

Currently, real-time PCR is most widely used and thus the standard method to quantify EBV-DNA. In the past, a “homemade” real-time PCR system was used in each facility, leading to considerable inter-laboratory biases. Commercially available kits are now more prevalent, providing reproducible results. Fully automated DNA extraction and amplification systems should promote the accuracy and speed of the assay while saving labor costs (56, 57). In the future, new technologies such as digital PCR, a novel method for the absolute quantification of target nucleic acids, may replace conventional real-time PCR.

## Author Contributions

HK and Y-LK contributed to the concept development process and to the writing and review of this manuscript. They also gave final approval of the version to be published.

### Conflict of Interest Statement

The authors declare that the research was conducted in the absence of any commercial or financial relationships that could be construed as a potential conflict of interest.
